# Pathological and immunohistochemical study of lethal primary brain stem injuries

**DOI:** 10.1186/1746-1596-7-54

**Published:** 2012-05-21

**Authors:** Sun Rongchao, Yang Shudong, Zhou Zhiyi

**Affiliations:** 1 Department of Pathology, Wuxi People’s Hospital Affiliated to Nanjing Medical University, 299 qingyang road, Wuxi, 214023, China

**Keywords:** Primary brain stem injury, Pathology, Immunohistochemistry, Differential diagnosis

## Abstract

**Background:**

Many of the deaths that occur shortly after injury or in hospitals are caused by mild trauma. Slight morphological changes are often found in the brain stems of these patients during autopsy. The purpose of this study is to investigate the histopathological changes involved in primary brain stem injuries (PBSI) and their diagnostic significance.

**Methods:**

A total of 65 patients who had died of PBSI and other conditions were randomly selected. They were divided into 2 groups, an injury group (25 cases) and a control group (20 cases). Slides of each patient’s midbrain, pons, and medulla oblongata were prepared and stained with HE, argentaffin, and immunohistochemical agents (GFAP, NF, amyloid-ß, MBP). Under low power (×100) and NF staining, the diameter of the thickest longitudinal axon was measured at its widest point. Ten such diameters were collected for each part of the brain (midbrain, pons, and medulla oblongata). Data were recorded and analyzed statistically.

**Results:**

Brain stem contusions, astrocyte activity, edema, and pathological changes in the neurons were visibly different in the injury and control groups (*P* < 0.05). Characteristic changes occurred in the neural axons, axon diameter varied from axon to axon and even over different segments of one axon, and several pathological phenomena were observed. These included segmental thickening and curving, wave-like processing, disarrangement, and irregular swelling. A few axons ruptured and intumesced into retraction balls. Immunohistochemical MBP staining showed enlargement and curving of spaces between the myelin sheaths and axons in certain areas. The myelin sheaths lining the surfaces of the axons were in some cases incomplete and even exfoliated, and segmentation disappeared. These pathological changes increased in severity over time (*P* < 0.05).

**Conclusions:**

These histopathological changes may prove beneficial to the pathological diagnosis of PBSI during autopsy. The measurement of axon diameters provides a referent quantitative index for the diagnosis of the specific causes of death involved in PBSI.

**Virtual Slides:**

The virtual slide(s) for this article can be found here: http://www.diagnosticpathology.diagnomx.eu/vs/1345298818712204

## Introduction

Brain stem injury is injury to the midbrain, pons, or medulla oblongata. It is a very serious form of brain injury and can be divided into two types: 1) primary brain stem injury, which is the result of the direct external violence, and 2) secondary brain stem injury, which is caused by other severe brain damage, such as brain herniation or cerebral edema [1]. Primary and secondary brain stem injuries differ in times of symptom onset and in physical signs. The symptoms and physical signs of secondary brain stem injury emerge gradually after the injury and death generally occurs later than in primary brain stem injury. In secondary brain stem injury, death cannot occur immediately after brain injury [2,3].

Primary brain stem injury (PBSI) makes up a large proportion of all recorded craniocerebral injuries and has a very high mortality rate. It includes brain stem transection, contusion hemorrhages, diffuse axonal injuries (DAI), and edema. The last three types of damage can be caused by minor trauma. Standard head checks do not reveal obvious skull fractures, subdural hemorrhages, epidural hematomas, or brain contusions [4].

Macroscopically, the morphological changes involved in these injuries are not usually obvious, but they can be lethal. Many of the deaths that occur shortly after injury or in hospitals are caused by mild trauma, and slight morphological changes can be observed in their brain stems during autopsy [5]. Because the original lesions and subsequent trauma coexist and affect each other, there is usually some question regarding the specific cause of each patient’s death [6]. This has consequences in both medical and juristic affairs. More and more researchers in pathology and clinical medicine have begun to pay attention to this issue. According to the literature, damage to axons and neurons is the major pathological cause of death in primary brain stem injury [7]. In our retrospective study, we obtained samples from multiple planes of the organs, cerebral lobes, and brain stems of 25 cadavers known to have died of blows to the head or face. We focused on the morphological changes involved in brain stem injuries and processed the samples using immunohistochemical methods and special staining, hoping to provide a pathomorphological foundation of diagnostic significance for diagnosis of causes of death in PBSI.

## Materials and Methods

### Materials

Two hundred and fifty autopsies were performed in our department between Jan. 1993 and Jun. 2008. Of these, 175 patients (70.0%) were entrusted to us by the national security bureaucracy, 68 (27.2%) were due to medical affairs, and only 7 involved medical research performed in our hospital. Of these cases, 46 (18.4%) involved cranial injury, and 25 of these (10.0%) showed definitive head and face trauma without skull fracture, subdural hemorrhage, epidural hematoma, or brain contusions as determined by routine examination. Nor was there any other visible cause of death, such as disease, intoxication, or trauma to other organs. For these 25 cases, no cranial bone fracture or definitive intracranial mass was found under routine testing. The diagnosis offered by histopathological examination during autopsy was primary brain stem injury. The ages of the patients ranged from 17 to 80 years at time of death, and the average age was 35. There were 22 male patients and 3 female patients. There were 14 cases of patients having been hit by a fist, 4 by palm, and 3 cases by other blunt injury. (The number of strikes suffered by the victim ranged from one to several.) In 2 cases, the patient had at least allegedly fallen to the ground, and the other 2 cases involved traffic accidents. Head injury occurred while moving in 21 cases (84%). Mild bruises and contusions were found in the head and face areas of 15 cases patients. Coma occurred within 1 hour of the injury in all cases—at once in 18 cases—followed by seizures, incontinence, dilation of pupils, respiratory inhibition, circulation disorders, and unconsciousness. All the victims died within 30 hours of injury, five within 5 min (3 of these immediately), seven in 10–30 min, five in 30 min to 4 h, six in 4–24 h, and two in 25–30 h. In the 40-case control group, 20 cases involved cardiovascular disease (control group 1) and 20 did not (control group 2, patients died of hemorrhagic shock, pneumonia, etc.).

### Methods

Samples from all the internal organs were collected using routine techniques. Coronary slides of brain tissue 1 centimeter thick were fixed and prepared for observation. Samples from each lobe of the cerebrum, hippocampus, and basal ganglion were collected as well. The horizontal method was used to dissect 2 to 3 tissue blocks from each part of brain stem, such as the midbrain (anterior to cerebral peduncle and posterior to colliculus caudalis) and the upper part of pons and medulla oblongata (anterior to basilar part and posterior to the wall of the fourth ventricle). The SP method was used to manipulate the immunohistochemical staining. All antibody reagents, such as GFAP monoclonal antibody, NF polyantibody, amyloid-ß monoantibody and MBP polyantibody, were bought from the Maixin Biological Development Company and used according to the manufacturer’s instructions. Axons were stained using immunohistochemical methods and the Bielschowsky-improved argentaffin method. Then NF-stained samples were observed under a light microscope at low power (×100). The diameter of each slide’s thickest longitudinal axon was measured by microscopic micrometer at its widest point, and 10 such diameters were collected for each part of the brain (midbrain, pons and medulla oblongata). The average of the 10 diameters was calculated as the value of the axon diameter for that part of the brain stem, and the data from the injury group and 2 control groups were analyzed statistically and compared. All specimens were handled and made anonymous according to the ethical and legal standards. The study was approved by the Ethical Committee of Wuxi People’s Hospital Affiliated to Nanjing Medical University, Wuxi, Jiangsu Province, China and was in compliance with the Helsinki Declaration.

### Statistical analysis

All data were processed using SPSS 11.5 software. The *χ*^2^ test was used to compare the proportions of two samples. Single-factor analysis of variance was used to compare the means of multiple samples. The Student-Newman-Keuls test was used to compare the means. The difference was considered to have statistical significance when *P* < 0.05.

## Results

### Gross observation of brain stem

In this sample group, there was no obvious abnormality in the gross view of the brain stems in 19 cases (76%), but scattered thin subarachnoid hemorrhage foci, with diameters of about 0.2 to 0.4 cm, were observed in 6 cases (24%). When the brain stem was viewed in cross-section, there were a few scattered, large, pinpoint hemorrhage foci near the edges of the ventral and dorsal sides were visible in 4 cases (16%). In the other 21 cases, only congestion of the cerebrum and cerebellum was visible. No abnormal phenomena were observed on the surfaces or in the cross-sections in the control group.

Histomorphological observation: The main pathological changes, susceptible areas, and incidence of brain stem injury are listed in Table 1.

**Table 1:** Distribution of morphological changes in 25 brain stems

	Region and Incidence		
Morphological Changes	Midbrain (%)	Pons (%)	Medulla Oblongata (%)
Superficial Contusion	13 (52.00)	8 (32.00)	7 (28.00)
Internal Contusion	21 (84.00)	18 (72.00)	14 (56.00)
Astrocyte Activity	14 (56.00)	7 (28.00)	12 (48.00)
Edema	4 (16.00)	6 (24.00)	14 (56.00)
Neuron Disorder	22 (88.00)	19 (76.00)	16 (64.00)
Hyperplasia of Microglia	15 (60.00)	9 (36.00)	13 (52.00)

### Morphological changes associated with brain stem injuries

The basic morphological changes observed in the brain stems were the same as those noted in primary brain stem injuries due to punching, slaps, blunt force, falls, and traffic accidents. In our 25 cases of primary brain stem injury, superficial brain contusions, such as hemorrhage around small vessels, congestion, edema, acute reaction of astrocytes, ischemic changes of neurons, and other pathological changes, were observed by microscope after routine HE staining of slides from the midbrain, pons, and medulla oblongata. Statistically significant differences (*P* < 0.05) appeared between the injury and control groups with respect to the incidences of these pathological changes. We conclude that recognition of the pathological changes outlined above may be beneficial to the diagnosis of brain stem injuries.

#### Contusions

Brain stem injury was usually observed at the area lateral to the cerebral peduncle, the fourth ventricle of the cerebrum, the pons, and the ventral part of medulla oblongata groove, but there were more sites of injury in marginal areas. These injuries were more obvious than those in internal areas. These changes included thin local subarachnoid hemorrhages. In a few cases, there was rupture of arachnoids or pia mater, small-granule disintegration of superficial tissue structure, tissue loosening, and edema. Hemorrhage was also present, showing patterns of scattered spotty bleeding, small amounts of focal bleeding or bleeding around the vessels, infiltration into brain tissue along the blood vessels, and congestion. However, in the control group, bleeding around the blood vessels, which appeared a scattered and irregular, was rare. This might indicate a transudatory hemorrhage caused by ischemia.

#### Astrocyte activity

**Figure 1:**
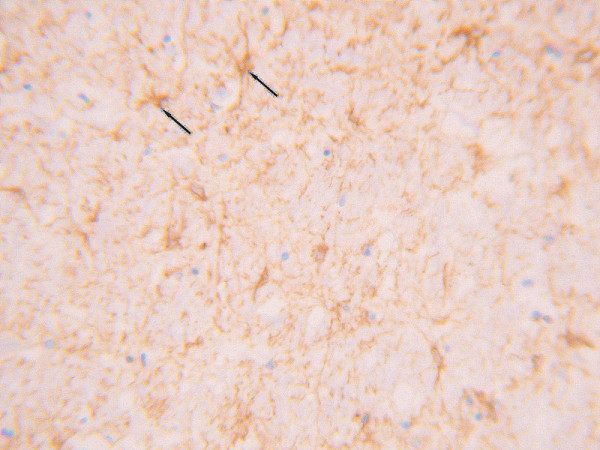
Under GFAP staining, the astrocytes in the contusion areas showed a growth pattern of focal hyperplasia with abundant cytoplasm, large cells, and darker cytoplasmic stains than non-contusion-area cells. (SP method, high power microscopic view).

Under immunohistochemical GFAP staining, astrocyte activity was observed in 1/3 to 1/2 of the brain stem samples in the brain stem injury group. Staining was found to be more common in the midbrain and medulla oblongata than in the pons. The astrocytes in the contusion areas showed a growth pattern similar to that of focal hyperplasia, with richer cytoplasm, larger cell size, and darker cytoplasmic staining. Cells gained more processes and took on different appearances. The population of cells became larger as time passed (Figure 1). In contrast, GFAP-positive cells were found to be sparse in the white matter, gray matter, and around the blood vessels in the brain stems of the control group. The astrocytes showed a star-ray appearance, with light staining of the cytoplasm, few branches, and long, thin processes.

#### Edema

The space around the vessels became larger and flocculated fluid was observed. The brain tissue around the vessels became looser, taking on a sponge-like appearance. These pathological changes gradually increased in severity from the midbrain to the medulla oblongata.

#### Neuron damage

The pathological changes observed in the neurons appeared as follows: Nissl bodies at the margins of the neurons dissolved and disappeared, the eosinophilic properties of cytoplasm changed, and karyopyknosis processes took on dark-stained triangle shapes with eosinophilic nucleoli.

#### Hyperplasia of microglia

The cells became more spherical as they transformed from the static state to the active state. The processes shortened and the nuclei became oval, rod-like, or triangular in shape.

#### Morphological changes in the neurons

**Figure 2:**
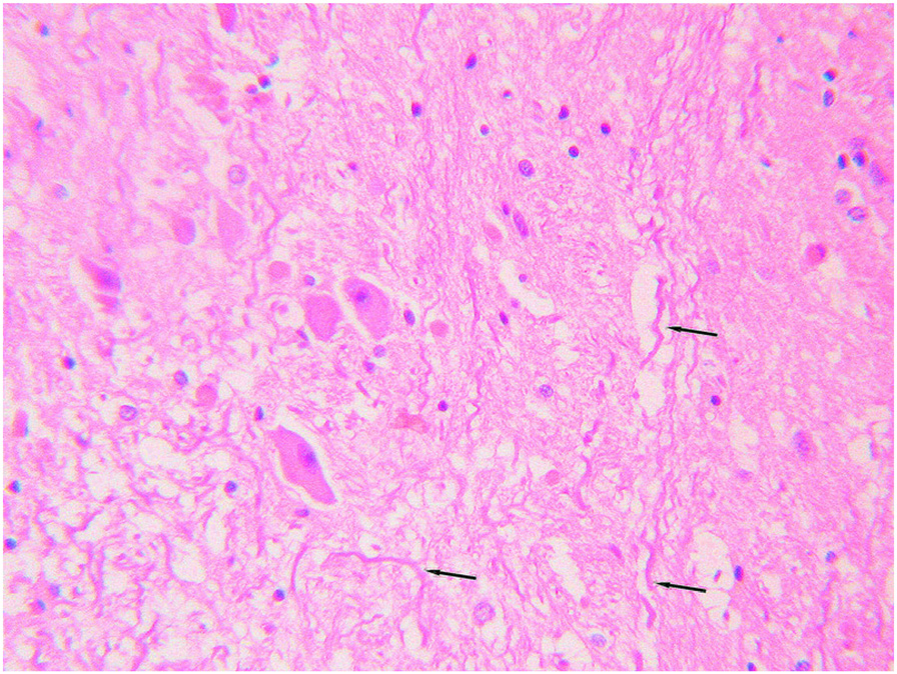
**In the injury group, axonal diameter varied from axon to axon and even between different segments of one axon.** The pathological phenomena observed included segmental thickening and curving, wave-like processing, derangement, and irregular swelling. (HE staining, moderate power microscopic view).

**Figure 3:**
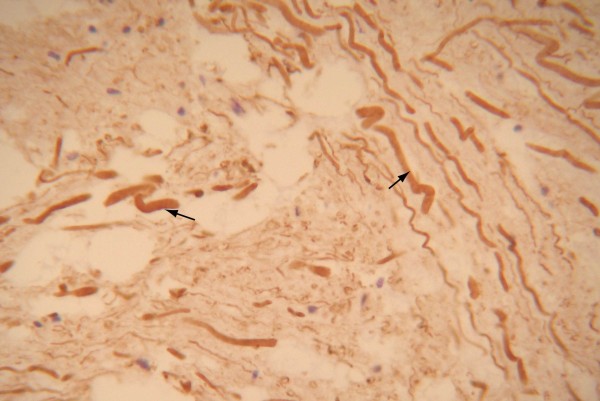
NF staining showed axon swelling and curvature. Some even formed a spiral pattern. (SP method, high power microscopic view).

**Figure 4:**
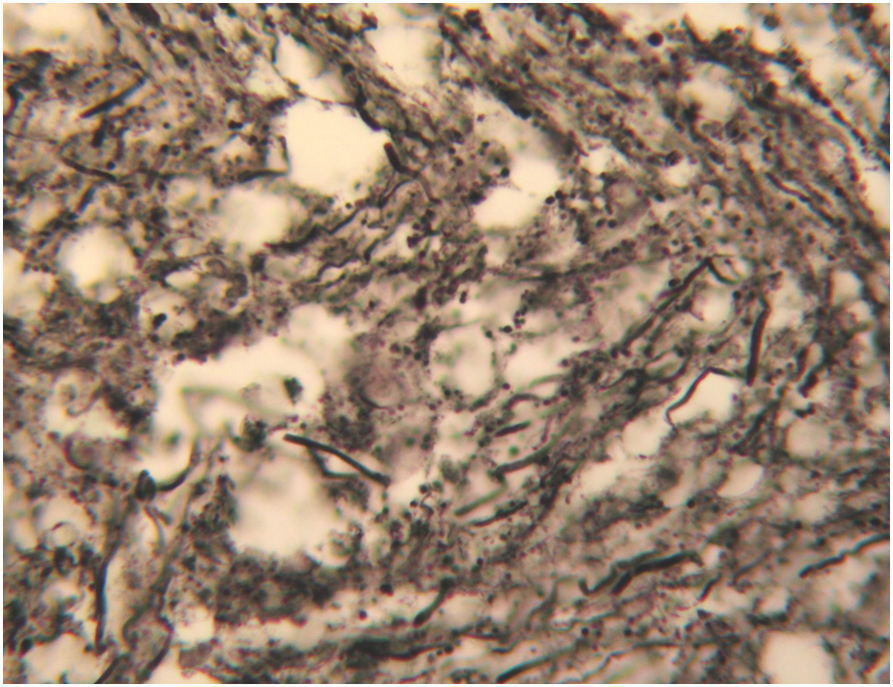
**Argentaffin staining showed axon swelling and curvature in the contusion region.** (Bielschowsky modified method, high power microscopic view).

**Figure 5:**
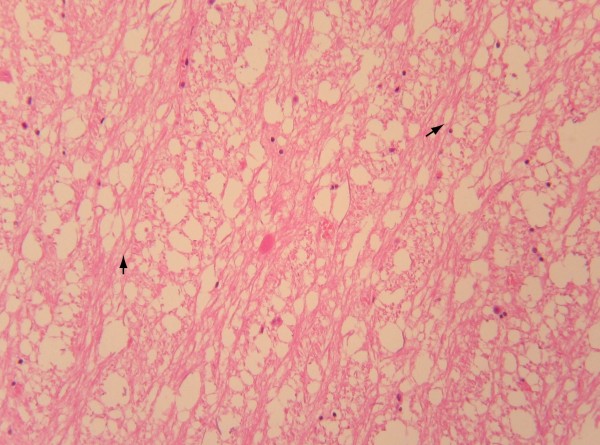
**In the control group, the axons maintained straight processes and showed little or no curvature and even diameters.** Irregular enlargement was rare. (HE staining, moderate power microscopic view).

**Figure 6:**
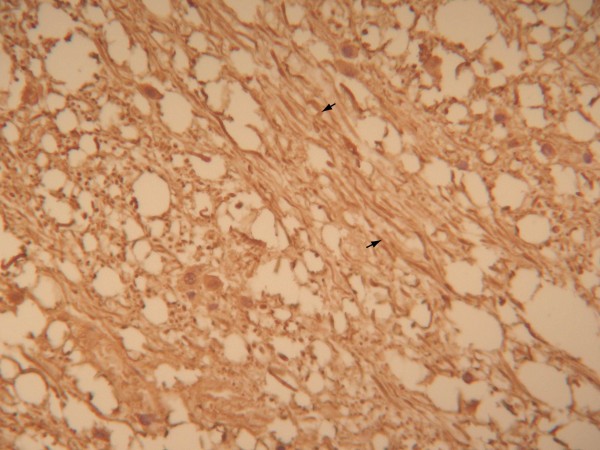
**NF staining showed a straighter and thinner pattern in the axons of the control group.** (SP method, moderate method, moderate power microscopic view).

**Figure 7:**
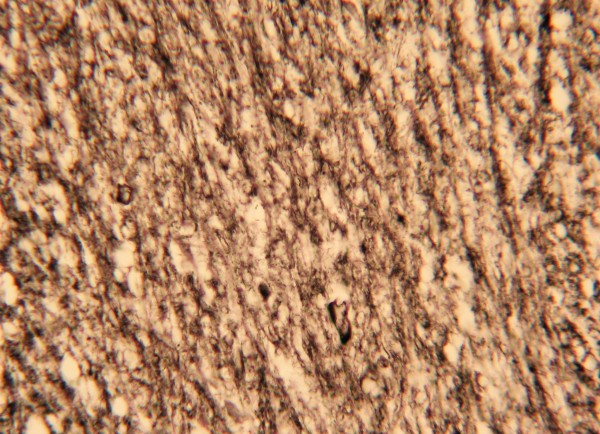
**Argentaffin staining showed a straighter and thinner pattern in the axons of the control group.** (Bielschowsky moderate method, moderate power microscopic view).

NF and HE staining of neuron axons were used to observe and measure relative indices using light microscopy. In the injury group, axon diameter varied from axon to axon and even over different segments of one axon. The pathological phenomena observed included segmental thickening and curving, wave-like processing, disarrangement, and irregular swelling. A few axons ruptured and intumesced into retraction balls (Figures 2 and 3). Immunohistochemical MBP staining showed enlargement and curving of spaces between the myelin sheaths and axons in certain areas. The myelin sheaths lining the surfaces of the axons were in some cases incomplete and even exfoliated, and segmentation disappeared. These pathological changes increased in severity over time. They were also apparent under argentaffin staining (Figure 4). In the control group, however, the axons maintained a pattern of straight processing, minimal curvature, and even diameter. Irregular enlargement was rarely observed (Figures 5, 6 and 7). The injury group showed significantly more axon swelling than the control group (*P* < 0.05) (Table 2). There were also 8 patients who showed positive expression (32%). They were visible under amyloid-ß staining.

**Table 2:** Analysis of axonal diameters (μm) measured in the injury group and control group

Group	Mean diameter of axons measured at each region (χ ± s)			Mean diameter of axons of the 3 regions (χ ± s)
	Midbrain (cases)	Pons (cases)	Medulla Oblongata (cases)	
Control Group 1	3.70 ± 0.70^*^(20)	3.95 ± 0.76^*^(20)	4.20 ± 0.73^*^(20)	3.95 ± 0.73^*^(20)
Control Group 2	3.64 ± 0.66^*^(20)	3.82 ± 0.69^*^(20)	4.01 ± 0.72^*^(20)	3.83 ± 0.68^*^(20)
Injury Group	4.73 ± 0.72(25)	4.78 ± 0.72(25)	5.23 ± 0.72(25)	4.91 ± 0.73(25)
Variance Analysis	*F* = 7.83, *P* < 0.05	*F* = 5.16, *P* < 0.05	*F* = 8.24, *P* < 0.05	*F* = 20.70, *P* < 0.05

* Relative to the injury group, *P* < 0.05.

### Morphological changes of the cerebrum and cerebellum

The only change that could be observed with the naked eye in was congestion in the cerebrum and cerebellum. Under a microscope, congestion of the pia mater and brain parenchyma, ischemic changes of the neurons, and mild edema became visible. There were no lethal lesions.

## Discussion

### Mechanism of brain stem injury

The clinical manifestations of primary brain stem injury include unconsciousness, decerebrate rigidity, disappearance of corneal reflex, and changes in vital signs such as respiration and heart rate. However, PBSI involves many more pathological morphological changes. This indicates a complicated pathogenesis [8]. Due to the particular anatomical shape and hidden position of the brain stem, it is usually difficult for blunt force to reach it directly. In the 25 cases in which force had been exerted on the head or face, all brain stems showed all of the above pathological changes. Brain stem injuries are caused by relative movement of the brain; the brain stem hits the clivus and free margin of the cerebellar tentorium and causes abrasions on the surrounding anatomical structures. This kind of injury is relatively mild, appearing as edema, focal contusions, and hemorrhage of the outer area and parenchyma of the brain stem. The factors that induce injury to the internal portions of the brain stem may include differences in the direction and velocity of the brain stem, cerebrum and cerebellum and movement or even shearing-force injury between each vessel and its surrounding semicolloid brain parenchyma. This mechanism may then lead to a regular process that manifests as edema, hemorrhage swelling, and rupture of axons [2,3].

### Changes visible under immunohistochemical staining and special staining (silver staining) with respect to brain stem injury

#### Glial fibrillary acidic protein (GFAP)

GFAP is characteristic of astrocytes. In experimental animal models, the number of GFAP-positive cells have been found to increase dramatically after brain stem injury [9-11]. In our study, GFAP-positive cell were found in great numbers. GFAP-positive cells of the brain stem injury group appeared darker under cytoplasmic staining, showed greater numbers of processes, and were more irregular in shape than control cells. All differences were found to be significant relative to the control group. This phenomenon indicates that an acute response from astrocytes can be clearly expressed in the superficial contusion region of the brain stem. This be considered a criterion in the diagnosis of brain stem injuries [12].

#### Neurofilaments (NF)

Large amounts of phosphorylated and non-phosphorylated NF, a neuronal structural protein, have been found in axons and dendrites [13,14]. Some researchers have used NF to study the changes in axons after brain injury. Local swelling and focal NF aggregation have been observed in neural axons within 1–2 h of injury, derangement within 6 h, and granular aggregation and obvious swelling within 72 h [5]. Axon rupture ends this process. We observed irregular swelling and rupture in axons after brain stem injury. External forces can induce neural axon rupture or NF derangement directly and indirectly. This can interrupt axoplasmic transfer and local aggregation of NF. Therefore, it is reasonable to use NF as an indicator of the morphological changes in the axons [15-17].

#### MBP

MBP is the major protein found in the myelin of the CNS and is of great importance to the formation of myelin sheaths and the neuron development [6]. Immunohistochemical testing has shown that MBP can be used to determine the changes in myelin in neurons after brain injury. Using these techniques, patent curvature, exfoliation, segmental disappearance, and other changes can be observed with respect to myelin. However, these changes occur later on in brain stem injury than the changes in the neural axons. In this way, MBP indicates that both axons and myelin undergo pathological changes after injury [18].

#### Amyloid-ß

With the help of immunohistochemical techniques, researchers have found amyloid-ß in the cortex after mechanical brain injury. Amyloid-ß exerts direct toxicity on neurons. This phenomenon also occurs in Down’s syndrome and Alzheimer’s disease [19]. We detected amyloid-ß in 32% of the neuronal axons under study. The mechanism of this is as yet undiscovered. The expression of amyloid-ß has certain referent value in the auxiliary diagnosis of PBSI [20].

#### Argentaffin staining

Argentaffin staining is the traditional method of observing neural axons, and it can also be used to indicate pathological changes in axons after injury. These include curvature, swelling, rupture, and the formation of contraction balls.

In conclusion, the results of these four kinds of staining methods are regular and relatively specific. This may make them suitable for the postmortem diagnosis of patients who die soon after acute brain stem injury.

### Diffuse axonal injury (DAI)

Typical histological changes in DAI include the formation of axonal balls and thickening, extension, and distortion of axons. Axon swelling and thickening and uneven swelling of the brain stem can be a manifestation of DAI and evidence of brain stem injury [21]. The concept of DAI, first introduced by Adams, involves brain injury induced by external force and characterized by profound axon changes in the brain. DAI has come to be considered an unique type of brain injury, one that can coexist with other types of cranial injury [22]. However, the pathogenesis of DAI is not thoroughly known. There is still controversy over whether the axon swelling and thickening observed in the brain stems of PBSI patients can be considered an independent clinical pathological entity [3]. We used NF immunohistochemical staining to detect the positively expressed substances in axons, then processed those data and compared them to those of the control group. Axon diameter in the brain stems of healthy people is 0.5–3 μm, which is shorter than the mean diameter (4.91 ± 0.73 μm) of our control group. This may be due to measurement error or postmortem changes. Significant differences were observed between the injury group and both the non-cardiovascular and cardiovascular control groups (*P* < 0.05). These differences indicate that the thickening and swelling of axons is related to the injury.

Axon diameter can serve as a quantitative referent for the diagnosis of cause of death in patients who die from brain stem injuries. Factors such as postmortem changes, ischemic changes, and anoxic changes should be taken into consideration during diagnoses in patients with PBSI. If several thickened axons are present, a diagnosis of diagnose axonal injury is inappropriate. Postmortem changes and the influence of other potential causes of death should be investigated when necessary. Only once this information is combined with other morphological changes involved in brain stem injury can an affirmative diagnosis of PBSI be made [2].

The pathomorphological and immunohistochemical changes observed in the brain stem after PBSI indicate that symptoms and deaths have objective material bases. This provides objective and scientific evidence suitable for the analysis and determination of injury and cause of death, determination of time of death, and determination of premortem vs. postmortem injury.

## Competing interests

The authors declare that they have no competing interests.

## Authors’ contributions

SR: participated in study design and coordination, material support for obtained funding, and supervised study; YS: participated in analysis and interpretation of data, carry out part of the experiments; ZZ: help to translated and edit the paper. All authors read and approved the final manuscript.

## Funding

This work was supported by a grant from Science and Technology Development Fund Key Project of Nanjing Medical University(Grant No.2011NJMU207).
